# Real-time surveillance of heat-related morbidity: Relation to excess mortality associated with extreme heat

**DOI:** 10.1371/journal.pone.0184364

**Published:** 2017-09-06

**Authors:** Robert W. Mathes, Kazuhiko Ito, Kathryn Lane, Thomas D. Matte

**Affiliations:** New York City Department of Health and Mental Hygiene, Queens, New York, United States of America; Georgia State University, UNITED STATES

## Abstract

The impact of heat on mortality is well documented but deaths tend to occur after (or lag) extreme heat events, and mortality data is generally not available for timely surveillance during extreme heat events. Recently, systems for near-real time surveillance of heat illness have been reported but have not been validated as predictors of non-external cause of deaths associated with extreme heat events. We analyzed associations between daily weather conditions, emergency medical system (EMS) calls flagged as heat-related by EMS dispatchers, emergency department (ED) visits classified as heat-related based on chief complaint text, and excess non-external cause mortality in New York City. EMS and ED data were obtained from data reported daily to the city health department for syndromic surveillance. We fit generalized linear models to assess the relationships of daily counts of heat related EMS and ED visits to non-external cause deaths after adjustment for weather conditions during the months of May-September between 1999 and 2013. Controlling for temporal trends, a 7% (95% confidence interval (CI): 2–12) and 6% (95% CI: 3–10) increase in non-external cause mortality was associated with an increase from the 50^th^ percentile to 99^th^ percentile of same-day and one-day lagged heat-related EMS calls and ED visits, respectively. After controlling for both temporal trends and weather, we observed a 7% (95% CI: 3–12) increase in non-external cause mortality associated with one-day lagged heat-related EMS calls and a 5% mortality increase with one-day lagged ED visits (95% CI: 2–8). Heat-related illness can be tracked during extreme heat events using EMS and ED data which are indicators of heat associated excess non-external cause mortality during the warm weather season.

## Introduction

Extreme summer heat has been consistently associated with excess morbidity and mortality in previous epidemiological studies[1–9]. Deaths due to hyperthermia (i.e., “heat-stroke”), an external cause, are a major fraction of deaths in U.S. that are attributed to weather events[10]. However, non-external cause deaths also increase during heat waves presumably due to the stress of heat on pre-existing conditions such as diabetes[11] and coronary heart diseases[12]. In a study of 107 U.S. cities, deaths due to cardiovascular, respiratory, and non-cardiorespiratory causes were all shown to be associated with heat waves[13]. Thus, non-external mortality impacts of extreme heat events are likely far larger than those manifested in hyperthermia deaths. In New York City, non-external cause deaths attributable to–extreme heat events have been estimated to be more than 10-fold greater in number than hyperthermia deaths[14].

Mortality data are generally not available for timely surveillance during extreme heat events. Emergency medical services (EMS) and emergency departments (ED) data, which have been used for near real time surveillance of influenza and other communicable diseases, have the potential to enable more timely surveillance of public health impacts during heat waves[15]. During periods of high temperature, calls to EMS have been reported to increase up to 10% over normal levels[3,9]. Visits to EDs also have been observed to increase[6], especially among the elderly[5].

Following a severe heat wave in 2006, syndromic surveillance of daily heat-related calls to EMS (determined by dispatchers) and heat-related visits to ED (based on chief complaint text) was implemented by the New York City (NYC) Department of Health and Mental Hygiene (DOHMH) in 2007 and is actively monitored in NYC during the warm weather months from May 1 to September 30. Data are analyzed daily during extreme heat events, when the city’s heat emergency plan is activated to monitor increases in heat related illness, inform heat emergency responses, and augment public warnings based on weather forecasts. In NYC, a heat emergency is declared when the National Weather Service (NWS) issues a heat advisory, which occurs when the heat index is forecast to reach at least 95°F for two or more consecutive days or 100°F for any period of time. These criteria are based on a prior study relating maximum heat index to excess mortality[16].

To the extent that non-external cause deaths are associated with–these extreme heat events, a prediction model can be developed to estimate an increase in non-external deaths given weather variables alone. However, if the syndromic surveillance data for heat-related EMS and/or heat-related ED visits are predictive of non-external cause of deaths above and beyond what available weather data can predict, then these surveillance data would be an additional timely indicator of mortality impacts of heat, which would help the agency contextualize augmented heat alert messages during extreme heat events that are particularly severe in magnitude and last for more than one day. Thus, our objective was to examine the predictive capability of the heat-illness syndromic surveillance data for non-external deaths.

## Methods

### Ethics statement

We did not submit this project to the NYC DOHMH Institutional Review Board, as the existing data are collected as part of routine public health surveillance, could not be linked to individuals, and were analyzed anonymously.

### EMS heat calls

Calls to the NYC EMS system are recorded and categorized into more than 250 unique call types from a drop-down menu, including heat-related complaints. Call type is determined by the dispatcher on duty and based on caller complaint, using a standardized telephone triage algorithm. Data files contain date and time of each call, call type, zip code, and hospital. EMS call data was available from 1999 to the time of data analysis, but we restricted the data analytical period up to year 2013 because that was the latest year for which mortality records were available. There were 7,149,489 NYC area EMS calls between May 1 and September 30, 1999–2013; 9,891 of these were coded as heat-related.

### ED data collection

At the time of the study, data from the EDs of 51 hospitals in NYC, composing approximately 98% of all ED visits in NYC, was sent to the DOHMH daily via direct file transfer. Electronic files contain date and time of each patient admission, age, sex, residential zip code, and the patient’s reason of visit or chief complaint, recorded as a free-text field. A text-processing algorithm scans the chief complaint field and classifies each visit into one of several syndromes. Details of the general methods used in this system have been published elsewhere[17]. Heat-related visits are identified by keywords such as “heat”, “hot”, “hyperthermia”, “heat stroke”, or “992”; the latter keyword is the International Classification of Diseases, Ninth Revision code for heat-related illness. We also include several keywords in the algorithm to exclude conditions unrelated to heat, such as those related to burns from appliances and hot drinks and food, conditions such as hot flashes, misspellings, et cetera. ED visit data were available from 2002 to the time of analysis, but, again, we restricted the data analysis period to 2013 to match availability of the mortality data. There were 17,650,447 visits to NYC EDs between May 1 and September 30, 2002–2013; 6,129 of these were classified as heat-related.

### Mortality data collection

Individual-level data on all deaths from all causes among NYC residents was obtained from the NYC DOHMH Bureau of Vital Statistics and coded according to the International Classification of Diseases, Tenth Revision for the years 1999–2013. This analysis used aggregate data on total number of daily deaths, total number of daily non-external cause deaths (ICD codes A00-R99), and date. A total of 293,803 non-external cause deaths were observed within the study period, from May 1 to September 30, between 1999 and 2013, and a total of 229,763 between May 1 to September 30, 2002 and 2013.

### Weather data collection

We used hourly meteorological data from LaGuardia Airport, one of three NYC weather stations maintained by the NWS, to compute daily weather variables for analysis because it had the most complete records of the three stations between May 1 and September 30, 1999–2013. Approximately 98% of hourly measurements of temperature were complete and 99.7% of daily estimates had at least 14 hourly measurements. These hourly measurements were used to derive daily maximum temperature and maximum heat index[18]. Temporal variation of temperature variables at LaGuardia Airport was highly correlated (0.93–0.97) with that from the other two NYC stations, Central Park and John F. Kennedy Airport. As we have done in our previous analysis of heat and mortality^18^, for the time-series analysis, the daily maximum heat index is the larger value of maximum heat index or maximum temperature, as heat index is not defined below 80°F.

### Data analysis

First, to describe the extent to which heat-illness indicators increase with extreme heat, we summarized heat-related EMS calls and ED visits during extreme heat days when near-real time heat illness surveillance is potentially most useful. For our analysis, we use the term “extreme heat event” (EHE) days to classify days that match the definition of NWS heat advisory criteria, described in the introduction, except that we used observed weather data rather than forecast data. Fifty-six extreme heat events (EHE), covering a total of 164 EHE days, occurred in NYC between May and September, 1999–2013. To capture lingering effects of temperature, as suggested by our previous analysis^18^, we included the three days following the end of each extreme heat event. Using this definition, there were a total of 304 EHE days.

Next, to assess whether heat-illness counts can predict mortality holding weather conditions constant, we developed time-series models of non-external cause mortality as a function of weather conditions and heat illness counts during the warm weather months of May to September. Our modeling approach was an extension of that used by the NYC DOHMH for surveillance of heat illness during heat waves. This automated daily analysis monitors heat-related EMS calls and ED visits from the previous day using data transmitted to the DOHMH overnight.

An over-dispersed Poisson generalized linear model (GLM)was fit to model non-external cause mortality in relation to the predictors of interest (EMS heat-related calls and ED heat visits), temporal, and meteorological variables and estimate the risk ratio (RR) and 95% confidence intervals (CI). Temporal variables included calendar date (centered to the middle of the warm season, July 16^th^, with values ranging from -76 (May 1) to 76 (September 30); July 16^th^ was centered to zero), a squared term for the centered date value, in addition to year, month, and holiday. Meteorological variables include linear, quadratic, and cubic terms for maximum temperature or heat index (whichever was higher) for zero to three day lags. We modeled lagged ED and EMS terms (0–3 days) with temporal and meteorological variables to assess their significance in the model (p<0.05 was considered significant based on the Wald chi-square test). The full model we tested was:

$$Mortality=\beta_{0}+\beta_{1}\left(DOW_{t}\right)+\beta_{2}\left(H\right)+\beta_{3}\left(Y_{t}\right)+\beta_{4}\left(M_{t}\right)+\beta_{5}\left(LT\right)+\beta_{6}\left(QT\right)+\beta_{7}\left(T\;linear_{lag}\right)+\beta_{8}\left(T\;quadratic_{lag}\right)+\beta_{9}\left(T\;cubic_{lag}\right)+\beta_{10}\left(Syndromic_{lag}\right),$$

where β_0_ is the model intercept, *DOW*_*t*_ is categorical day of week, *H* is a holiday indicator, *Y*_*t*_ is categorical year, *M*_*t*_ is categorical month, *LT* is linear time trend, *QT* is quadratic time trend, *T linear* is linear maximum temperature or head index, for a specific lag, *T quadratic* is quadratic maximum temperature or heat index, for a specific lag, *T cubic* is cubic temperature or heat index, for a specific lag, and *Syndromic* is heat-related EMS calls or ED visits for a specific lag. We also used an analysis of deviance test to determine whether the models with the ED or EMS variables showed better predictions than the models without them. First order residual autocorrelations were examined. We used SAS (version 9.2, SAS Institute, Cary, NC) for the parametric models. For comparison, we used penalized splines[19], as implemented for generalized additive models (GAM) in statistical package R (version 3.0.0, R Development Core Team) to fit temporal and meteorological terms using optimum degrees of freedom. The fitted values from the generalized additive model were used to estimate the shape of the relationship between heat-related EMS calls and ED visits and non-external cause mortality:

$$Mortality=B_{0}+ns\left(Time_{t},5\right)+ns\left(DOW_{t}\right)+s\left(T_{lag}\right)+s\left(Syndromic_{lag}\right),$$

where β_0_ is the model intercept, *Time*_*t*_ is the natural cubic spline of time with 5 degrees of freedom per year, *DOW*_*t*_ is the natural cubic spline of day of week with 6 degrees of freedom, *T*_*lag*_ is the spline of maximum temperature or heat index for a specific lag, and *Syndromic*_*lag*_ is the spline of heat-related EMS calls or ED visits for a specific lag.

We did not specifically model potentially larger impacts of EHE days that occur earlier in the summer because our previous analysis[16] showed little evidence of this in NYC.

**Fig 1:**
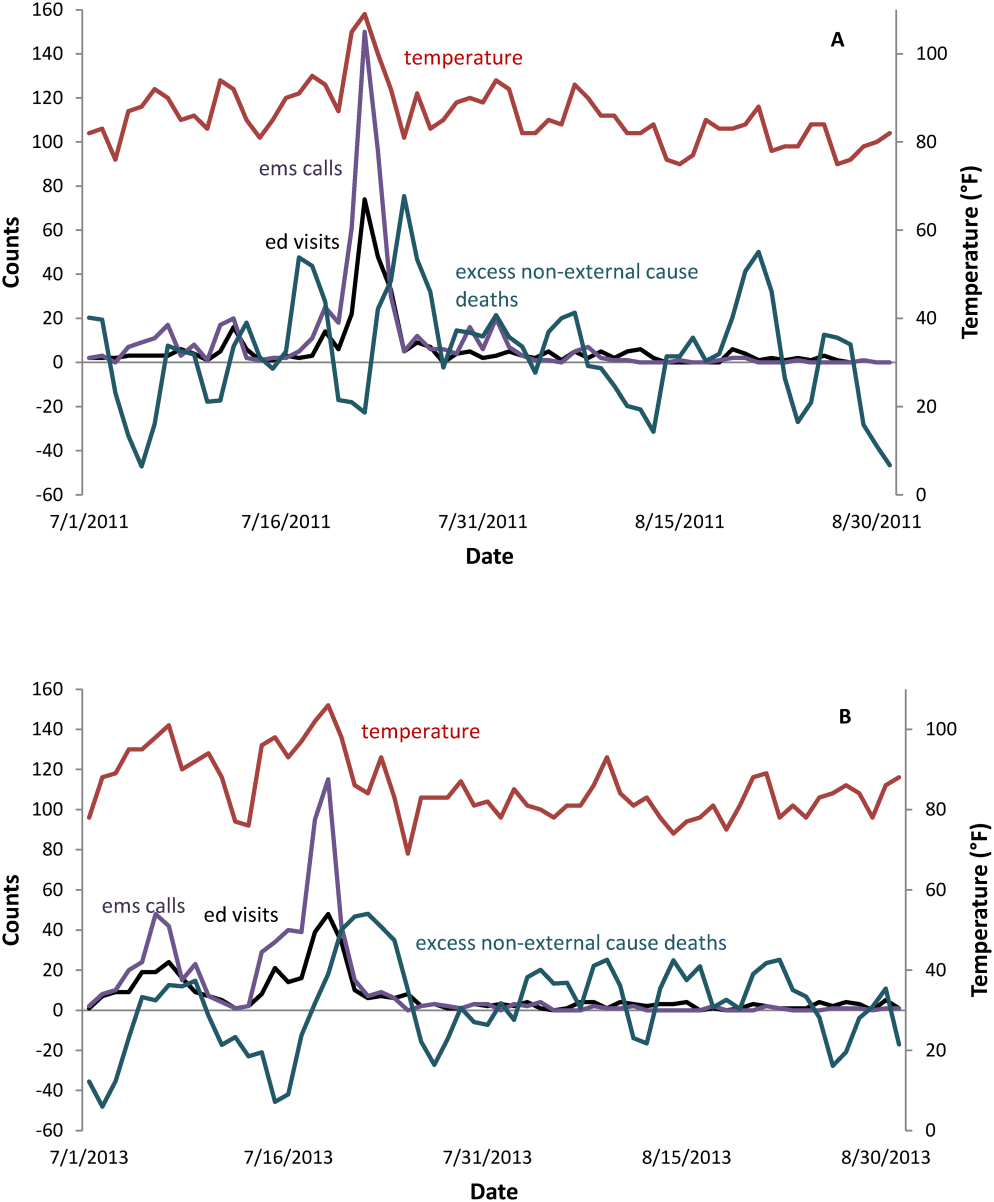
Time-series of daily heat-related EMS calls, ED visits, excess non-external cause deaths, and maximum temperature or heat index. (A) July-August 2011. (B) July-August 2013.

Finally, we estimated excess non-external cause mortality for Fig 1A and 1B by subtracting the expected mortality derived from the above temporal-only GLM from the observed daily mortality count.

### Results

Table 1 provides a summary of the syndromic heat-related indicators, temperature, and mortality data during EHE and non-EHE days. We observed a moderate increase in mean total calls to EMS and a decrease in mean total visits to EDs during the 56 observed EHE in NYC between 1999 and 2013. Both EMS and EDs experienced an increase in heat-related incidents during EHE days though the increase in heat-related EMS calls was much greater. The most heat-related EMS calls and ED visits in one day was 170 and 74, respectively. In contrast, only a modest increase in mean non-external cause deaths was observed during the EHE days. The mean maximum temperature/heat index at LaGuardia International Airport during the study period was 81°F with a maximum high temperature/heat index of 110°F. The majority of EHE days occurred in July (56%) and a large proportion (43%) occurred in the years 2002 and 2013. The mean maximum temperature/heat index during the 56 EHE was 98.5°F. When comparing daily trends in EHE periods, we found a larger increase in total EMS call volume during weekdays (8.6%) compared to weekends (3.3%).

**Table 1:** Daily mean counts and range of EMS calls (1999–2013), ED visits (2002–2013), and non-external cause deaths (1999–2013) during non-extreme heat event (EHE) and EHE days between May and September.

	Non- EHE days (n = 1986)			EHE days (n = 164)			EHE days + 3 day lag (n = 304)		
Heat-illness indicator	Daily Mean (SD)	Daily Minimum	Daily Maximum	Daily Mean (SD)	Daily Minimum	Daily Maximum	Daily Mean (SD)	Daily Minimum	Daily Maximum
EMS Heat Calls	1.9 (3.9)	0	65	31.9 (29.2)	1	170	20.3 (25.4)	0	170
EMS Total Calls	3116.5 (651.1)	1348	4310	3368.6 (734.8)	1524	4763	3296.4 (724.2)	1481	5039
ED Heat Visits	2.3 (2.2)	0	22	14.5 (13.5)	1	74	10.0 (11.4)	0	74
ED Total Visits	9635.3 (1749.0)	5558	17349	9415.0 (1765.7)	5738	12642	9485.8 (1794.3)	5738	12820
Non-external cause deaths	127.5 (14.4)	77	186	132.5 (16.8)	98	212	131.6 (17.8)	92	248
Maximum heat index	78.9 (8.7)	53	99	98.5 (4.1)	90	110	92.3 (8.2)	71	110

Fig 1A and 1B display the daily counts of heat-related EMS calls, ED visits, non-external cause deaths, and maximum heat index for two particularly hot summers in NYC. During the summer of 2011, maximum temperatures reached over 100°F for three of four consecutive days. Peaks in EMS calls and ED visits clearly coincide with the heat wave, with a slightly lagged peak of excess mortality. We also show an extended period of extreme heat during the summer of 2013, with seven straight days over 90°F. The effect on EMS calls and ED visits was slightly less dramatic, but peaks of these indicators do line up well with days when temperature or heat index is high. Again, excess mortality peaks several days after the extreme heat event.

In the temporal-only adjusted GLMs, goodness-of-fit statistics were similar between models that included lags 0–1 of EMS calls and ED visits and the full models (lags 0–3), but the analysis of deviance indicated that the fits for the models with EMS or ED variables were significantly better than those for the models without these morbidity indicators (Table 2). In the GLMs adjusted for both time and weather, terms for heat-related EMS calls and ED visits lagged one day only were found to be significant. Goodness-of-fit statistics were comparable between the more parsimonious model (lag 1 only) and the full models (lags 0–3). The models with EMS or ED variables lagged one day showed significantly better prediction than the models without these morbidity indicators. Heat-related EMS calls generally predicted non-external cause deaths better than ED syndrome data did.

**Table 2:** Comparison of fit for models of the relation of heat-related EMS calls and ED visits and non-external cause mortality.

	Parametric models				Nonparametric models			
Model parameters	DF	Deviance explained	Analysis of deviancep-value*	Correlation of raw and predicted values on HI ≥ 90°F days	DF	Deviance explained	Analysis of deviancep-value*	Correlation of raw and predicted values on HI ≥ 90°F days
**Temporal only models: EMS**								
Temporal trends only, 1999–2013	28	33.1		0.57	81	35.3		0.60
Temporal trends + EMS heat calls, lags 0–3	32	40.2	<0.001	0.69	85	40.7	<0.001	0.73
Temporal trends + EMS heat calls, lags 0–1	30	39.0	<0.001	0.70	83	40.4	<0.001	0.74
**Temporal only models: ED**								
Temporal trends only, 2002–2013	25	25.2		0.52	66	26.9		0.54
Temporal trends + ED heat visits, lags 0–3	29	27.9	<0.001	0.60	70	30.4	<0.001	0.63
Temporal trends + ED heat visits, lags 0–1	27	27.9	<0.001	0.60	68	30.1	<0.001	0.63
**Temporal + Meteorological models: EMS**								
Temporal trends only, 1999–2013	28	33.1		0.57	81	33.8		0.58
Temporal trends + maximum heat index, lags 0–3, 1999–2013	40	37.8	<0.001	0.66	85	40.6	<0.001	0.69
Temporal trends + maximum heat index + EMS heat calls, lags 0–3, 1999–2013	44	41.7	<0.001	0.70	89	41.8	<0.001	0.73
Temporal trends + maximum heat index + EMS heat calls, lag 1, 1999–2013	41	39.9	<0.001	0.71	86	41.4	<0.001	0.73
**Temporal + Meteorological models: ED**								
Temporal trends only, 2002–2013	25	25.2		0.49	66	27.4		0.55
Temporal trends + maximum heat index, lags 0–3, 2002–2013	37	29.7	<0.001	0.59	70	32.1	<0.001	0.60
Temporal trends + maximum heat index + ED heat visits, lags 0–3	41	30.4	0.09	0.60	74	32.5	<0.001	0.63
Temporal trends + maximum heat index + ED heat visits, lag 1	38	30.2	0.01	0.60	71	32.0	<0.001	0.62

*Analysis of deviance was conducted to compare the models with the weather, ED, or EMS variables vs. the models without them.

We obtained similar results from the non-parametric GAMs. Deviance explained, first order residual autocorrelation, and correlation of raw and predicted values on days with a heat index ≥ 90°F were found to improve with the addition of heat-related EMS calls and ED visits for temporal-adjusted models. Goodness-of-fit improved slightly with the addition of these terms to the models adjusted for both temporal trends and weather.

**Fig 2:**
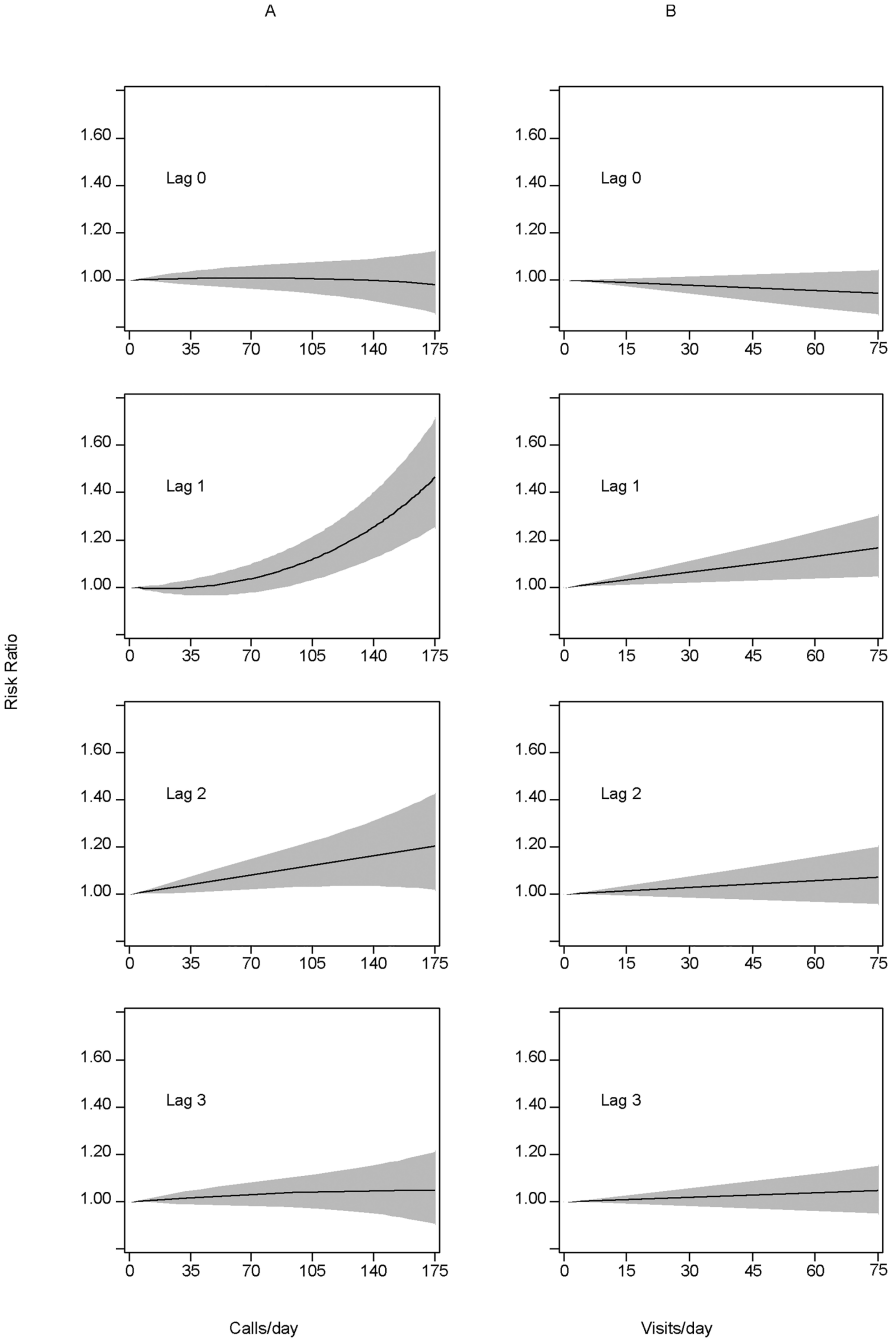
Relationship between non-external cause mortality lagged 0–3 days and heat-related (A) EMS calls and (B) ED visits for the weather-adjusted general linear models. Counts of heat-related EMS calls and ED visits are on the x-axis and risk ratio and 95% confidence interval are on the y-axis.

We first fitted the full GLM models (lags 0–3) of the relationship between heat-related EMS calls and ED visits to non-external cause mortality, controlling for temporal trends. A 7% (95% confidence interval (CI): 2–12) and 6% (95% CI: 3–10) increase in non-external cause mortality was associated with an increase from the 50^th^ percentile to 99^th^ percentile of same-day and one-day lagged heat-related EMS calls and ED visits, respectively. After fitting the full models adjusting for both temporal trends and weather (Fig 2), risk of non-external cause mortality increased 7% (95% CI: 3–12) with an increase from the 50^th^ percentile of one-day lagged heat-related EMS calls (n = 1) to the 99^th^ percentile (n = 60). At an increase from the 50^th^ percentile of one-day lagged heat-related ED visits (n = 2) to the 99^th^ percentile (n = 25), risk of non-external cause mortality increased 5% (95% CI: 2–8). We chose this range rather than the inter-quartile range given the distribution of heat-related EMS calls and ED visits were highly skewed. This skewness occurs because heat-related EMS calls and ED visits are so specific to high temperature that these types of calls and visits are almost never seen until the maximum temperature is above 90° F. The addition of terms for heat-related EMS calls and ED visits improves both the temporal-only adjusted model and temporal plus weather adjusted model. However, weather accounts for much of the increase in mortality until very high temperatures, and consequently also results in high numbers of heat-related EMS calls and ED visits. For example, the excess risk of mortality at the 99^th^ percentile of temperature (103°F) is 7% (95% CI: 5–11), compared to a 7% increase at the 99^th^ percentile of EMS calls and a 5% increase at the 99^th^ percentile of ED visits.

**Fig 3:**
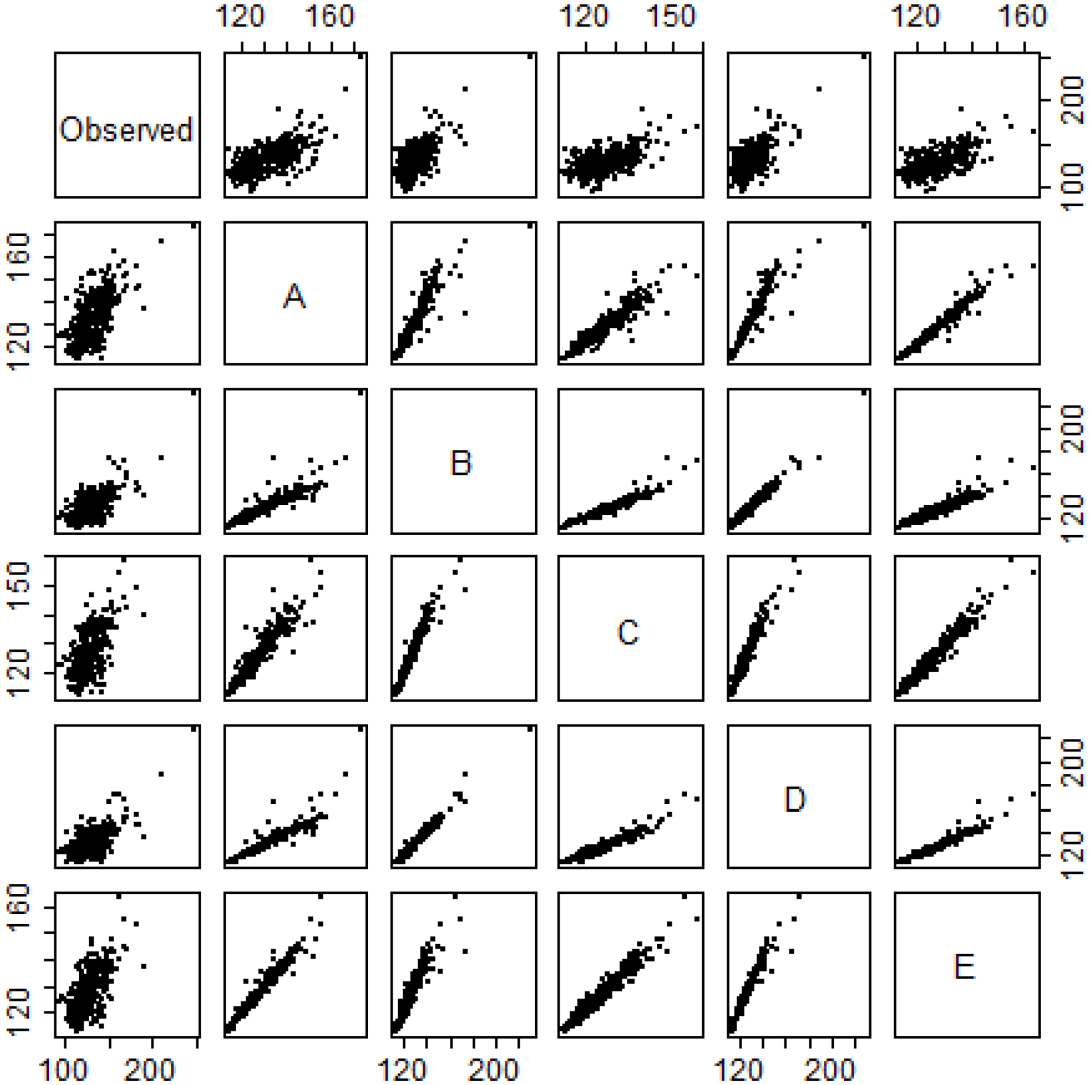
Scatterplot of observed non-external cause mortality and predicted counts from models where HI> = 90°F. (A) temporal and weather-adjusted model; (B) heat-related EMS calls, temporal-adjusted model; (C) heat-related ED visits, temporal-adjusted model; (D) heat related EMS calls, temporal and weather-adjusted model; (E) heat-related ED visits, temporal and weather-adjusted model.

**Fig 4:**
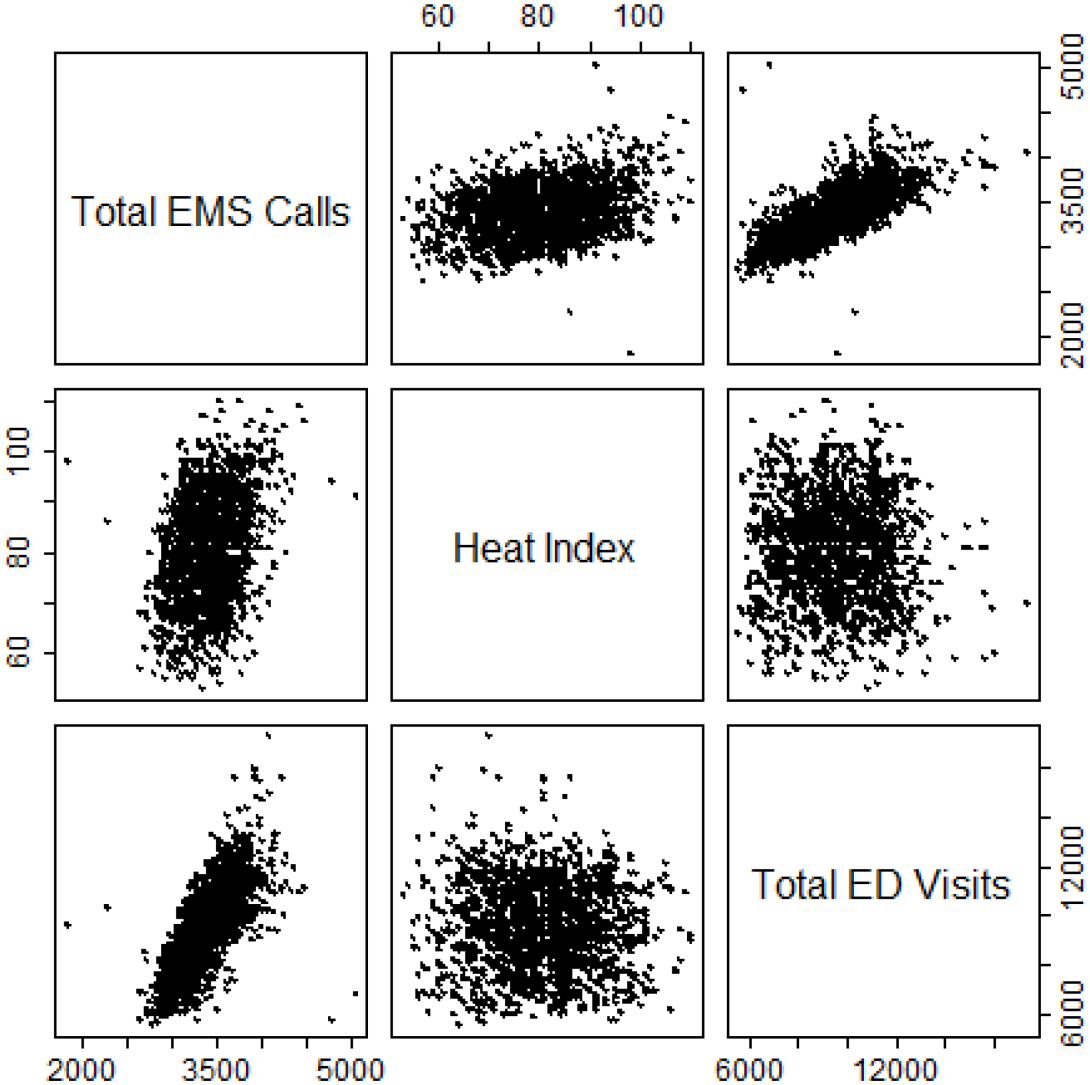
Scatterplot of total EMS calls, total ED visits, and maximum heat index.

We observed heat-related EMS calls and ED visits to be most predictive during extended EHE days. During a severe extreme heat event in NYC between July 21 and July 24, 2011, when the recorded maximum temperature/heat index for each day was between 92°F and 109°F, between 30 and 150 heat-related EMS calls and 22 to 74 heat-related ED visits were recorded per day. Based on the weather-only model, with no terms for EMS calls or ED visits, a 1 to 15% increase in non-external cause mortality would be expected. With the addition of EMS calls to the model, we estimate a statistically significant 2 to 43% increase in risk of non-external cause mortality during that period and a 6 to 18% increase in risk of non-external cause mortality with the addition of ED visits. Correlation between the predicted and observed non-external cause deaths on days with a heat index ≥ 90°F was observed to be high for our models (Fig 3). We note that rising maximum heat index modestly increased total calls to EMS and modestly decreased total visits to the ED (Fig 4).

### Discussion

Heat-related EMS calls, and to a lesser extent, heat-related ED visits, significantly predicted non-external cause mortality above and beyond that predicted by the heat index variable in NYC during the months between May and September from 1999 to 2013. We explored two model types, one controlling for temporal trends only and the other controlling for both temporal trends and weather. Maximum heat index was used as our primary weather metric, based on a previous analysis of non-external cause mortality in NYC that showed heat index gave the best model fit compared to other weather metrics[16]. To our knowledge, this study is the first to quantify the association between heat-related morbidity and non-external cause mortality using EMS and ED data.

Heat-related EMS calls on the same day and lagged one day were observed to predict non-external cause mortality in our temporal-adjusted model and EMS calls lagged one day predicted non-external cause mortality in our temporal and weather-adjusted model. In particular, risk of mortality rapidly increased as the number of heat-related EMS calls approached high levels (>100 calls/day) in both models. The parametric model that included heat index provided a significantly better fit compared to the model that included long and short-term temporal trends only. This is similar to the finding by Metzger, et al, which observed a lagged non-linear relationship between mortality and heat index >95°F. The magnitude of the relationship between mortality and heat-related EMS calls was strongest with heat-related EMS calls lagged one day. Our results suggest the addition of heat-related EMS calls to the model predicts mortality modestly above and beyond that of weather, especially during extreme heat waves that exhibit high temperatures and high numbers of EMS calls. Similar results were observed with our modeling of heat-related ED visits.

Adding terms for heat-related EMS calls and ED visits modestly improved model fit compared to the temporal and weather-adjusted model. The effect was more pronounced with the temporal only models, suggesting lagged EMS calls and ED visits are capturing the non-linear lagged high temperature effects. Same-day EMS calls and ED visits showed a linear relationship to mortality in our temporally-adjusted models, while lagged values had a non-linear relationship, especially at higher levels of EMS calls. After controlling for weather, the same-day association disappeared. We note the parametric and nonparametric models fit the data similarly well, confirming our parametric models are adequately fitting potential non-linear relationships.

During extreme heat events days, we observed an increase in total EMS calls compared to non-EHE periods. Weekdays in particular had larger increases in total calls in EHE periods versus weekends. Previous studies showed increases of 4–17% in total EMS calls during heat waves compared to normal or non-heat wave periods[3,9,20] and temporal differences in call patterns were also similar to what we observed. These observations may suggest a possible behavioral component to how individuals approach an EHE as people may be more likely to stay indoors on the weekend. For ED visits, we found a slight decrease in total visits during EHE periods compared to non-EHE periods. Our findings agree with a European study that showed no increase in total ED visits during a heat wave in the summer of 2006 except among those ≥75 years of age[5]. However, other studies[6,20,21] have found an overall increase in all-cause related ED visits during heat waves. A study of a 2006 California heat wave observed a significant 3% increase in risk of all-cause ED visits during the heat wave compared to reference periods before and after the heat wave[6]. Though our results do not agree with this latter study, ED visits from only one major heat wave were analyzed and the duration and severity of this heat wave was likely greater than a typical heat wave in NYC. Our observation of no all-cause increase in ED visits may be due to fewer people using the ED for ‘discretionary’ care, i.e. non-emergency medical needs, during hot weather. This finding suggests that during typical extreme heat events in NYC, EDs should generally expect no surge in total visits while EMS should expect a modest surge in calls. We should note that population increases and decreases would be expected in the summer months, either an influx of tourists or NYC residents leaving for vacation. However, we would not anticipate the increase and decrease to fluctuate in sync with short-term variations of temperature and their health impacts, and thus would not expect such variation to impact our model or conclusion.

This study has several strengths. First, 15 years of weather and health outcome data for the largest city in the U.S., where the impacts of–extreme heat events have been repeatedly shown in the past, provided high statistical power for the analysis. Secondly, because the EMS calls and ED syndromic data are monitored daily as part of NYC’s syndromic surveillance system, the findings from this analysis reinforce the value of using syndromic surveillance as an indicator of public health risk during heat waves. The result from this study should also be useful for other cities that have syndromic surveillance systems or plan to develop such systems.

This study also has a few weaknesses. Because EMS heat calls and ED heat visits were identified with very different methods (dispatcher interpretation of caller complaint vs. text search in ED visits chief complaint log), differences in their association with mortality may reflect different misclassification rates. For example, our text processing algorithm for heat-related visits to the ED identifies “heat-related” visits on cold weather days usually as a result of a misspelled word or wording mistake in the chief complaint field. Additionally, prior analyses show that heat-related ED visits tend to affect a younger population than heat-related hospital admissions and deaths[22]. Thus, syndromic surveillance may reflect different heat exposure settings and populations compared to those at risk of death or hospital admission. Our model did not take into account the sequence of heat waves, nor did we analyze sub-populations that might be disproportionately affected.

Nonetheless, the current heat surveillance system in NYC is useful in providing a timely indicator of the severity of heat waves in relation to mortality. We found heat-related EMS calls and ED visits to be a significant, though modest, predictor of non-external cause mortality during hot weather in NYC. However, it should be stressed that syndromic systems are complimentary to temperature when conducting risk assessment or informing a response, as forecasted weather continues to be the most important and timely measure when assessing mortality risk. In weather situations where there are complicating factors, such as a blackout during a period of extreme heat, syndromic data may become more important, not only in better predicting mortality, but also providing better situation awareness of morbidity.
